# DNAJA4 suppresses epithelial-mesenchymal transition and metastasis in nasopharyngeal carcinoma via PSMD2-mediated MYH9 degradation

**DOI:** 10.1038/s41419-023-06225-w

**Published:** 2023-10-24

**Authors:** Qun Zhang, Ping Feng, Xun-Hua Zhu, Shi-Qing Zhou, Ming-Liang Ye, Xiao-Jing Yang, Sha Gong, Sheng-Yan Huang, Xi-Rong Tan, Shi-Wei He, Ying-Qing Li

**Affiliations:** 1 Department of Radiation Oncology, The First Affiliated Hospital of Sun Yat-sen University, Guangzhou, 510080 PR China; 2 State Key Laboratory of Oncology in South China, Guangdong Key Laboratory of Nasopharyngeal Carcinoma Diagnosis and Therapy, Guangdong Provincial Clinical Research Center for Cancer, Sun Yat-sen University Cancer Center, Guangzhou, 510060 PR China

**Keywords:** Head and neck cancer, Head and neck cancer

## Abstract

Emerging evidence indicates that DNA methylation plays an important role in the initiation and progression of nasopharyngeal carcinoma (NPC). DNAJA4 is hypermethylated in NPC, while its role in regulating NPC progression remains unclear. Here, we revealed that the promoter of DNAJA4 was hypermethylated and its expression was downregulated in NPC tissues and cells. Overexpression of DNAJA4 significantly suppressed NPC cell migration, invasion, and EMT in vitro, and markedly inhibited the inguinal lymph node metastasis and lung metastatic colonization in vivo, while it did not affect NPC cell viability and proliferation capability. Mechanistically, DNAJA4 facilitated MYH9 protein degradation via the ubiquitin-proteasome pathway by recruiting PSMD2. Furthermore, the suppressive effects of DNAJA4 on NPC cell migration, invasion, and EMT were reversed by overexpression of MYH9 in NPC cells. Clinically, a low level of DNAJA4 indicated poor prognosis and an increased probability of distant metastasis in NPC patients. Collectively, DNAJA4 serves as a crucial driver for NPC invasion and metastasis, and the DNAJA4-PSMD2-MYH9 axis might contain potential targets for NPC treatments.

## Introduction

Nasopharyngeal carcinoma (NPC) is a malignant tumour occurring in the nasopharynx, and exhibits a characteristic geographical distribution, with the highest incidence in South China and Southeast Asia [1, 2]. This unique geographical distribution is attributed to both genetic and environmental factors [3, 4]. Due to the unique anatomical location and high radiosensitivity of NPC, radiotherapy is the mainstay treatment for NPC patients. The addition of chemotherapy to radiotherapy has greatly improved the survival rate of NPC patients; however, distant metastasis remains the main reason for treatment failure [5–7]. Thus, exploring the regulatory mechanism underlying NPC metastasis is essential for developing novel treatment strategies.

Heat shock proteins (HSPs), heat stress proteins that are widely expressed and produced under stimulation by high temperature, infection or hypoxic conditions, can increase the emergency response ability of cells to various stimuli [8]. In addition, HSPs play important roles in regulating cancer cell survival, invasiveness and dissemination, thus promoting the initiation and progression of tumours [9–13]. DNAJA4 (DNAJ Heat Shock Protein Family (HSP40) Member A4) is a member of the HSP40/DNAJ family and is involved in multiple biological processes [14, 15]. For instance, DNAJA4 can regulate the expression of F-actin and related pathway proteins in response to hyperthermia in keratinocyte HaCaT cells [16]. DNAJA4 deficiency promotes the transcription of TNF-α and IL-1B to activate NF-κB signalling, and thereby inhibits keratinocyte cell proliferation and viability during hyperthermic stimulation [17]. DNAJA4 deficiency can also protect keratinocytes from hyperthermia-induced injury by activating Clusterin and ERK signalling [18]. DNAJA4 participates in cholesterol biosynthesis as a molecular chaperone regulated by SREBP [19]. Interestingly, as a direct downstream effector of miR-199a and miR-1908, DNAJA4 negatively regulates metastasis, as well as endothelial recruitment and colonization, in melanoma [20]. However, the functions and mechanisms of DNAJA4 in NPC remain unknown.

Here, based on genome-wide methylation profiling, we reveal that hypermethylation of the DNAJA4 promoter causes downregulation of DNAJA4 expression in NPC. Overexpression of DNAJA4 suppresses NPC cell migration, invasion, epithelial-mesenchymal transition (EMT) and metastasis in vitro and in vivo. Mechanistically, DNAJA4 recruits PSMD2 to promote the degradation of the MYH9 protein through the ubiquitin-proteasome pathway. Restoration of MYH9 expression can rescue the DNAJA4-mediated suppressive effect on metastasis. Clinically, low expression of DNAJA4 is associated with poor prognosis in NPC patients. Collectively, our findings indicate that the DNAJA4-PSMD2-MYH9 axis contains potential targets for antimetastatic treatment of NPC.

## Materials and Methods

### Public datasets and bioinformatic analysis

Microarray data based on the Infinium Human Methylation 450 K BeadChip (GSE52068) were analysed to identify differentially methylated CpG sites of DNAJA4 in 24 paired NPC and normal nasopharynx tissues, and these sites were validated in the Hong Kong dataset (GSE62336). RNA sequencing (RNA-seq) data of 113 NPC tissues were obtained from an NCBI GEO dataset (GSE102349). The NPC tissues were divided into a high DNAJA4 expression group and a low DNAJA4 expression group. Gene set enrichment analysis (GSEA) was performed using GSEA version 4.2.3 software (https://www.gsea-msigdb.org/gsea/msigdb/index.jsp). Statistical significance was determined by comparing the enrichment scores generated from enrichment analysis of the gene set with 1000 permutations to calculate *p* values.

### Clinical specimens

A total of 25 normal nasopharynx and 40 NPC fresh frozen biopsy tissues were obtained from the Sun Yat-sen University Cancer Center (Guangzhou, China) for DNA, RNA or protein extraction. Another 212 paraffin-embedded NPC samples obtained between Jan 2004 and Jan 2007 were collected from the Sun Yat-sen University Cancer Center for prognostic analysis. No patient had received any antitumour therapy before biopsy sampling. The Institutional Ethical Review Boards of the Sun Yat-sen University Cancer Center approved this study (B2022-784-01) and waived the requirement to obtain written informed consent.

### Cell culture and treatment

The human normal nasopharyngeal epithelial cell line NP69 was incubated in keratinocyte serum-free medium (KSFM; Gibco, NY, USA). Human NPC cell lines (CNE1, CNE2, HNE1, C666-1, HONE1, SUNE1) were cultured in RPMI-1640 (Gibco) medium supplemented with 10% foetal bovine serum (FBS; ExCell Bio, Taicang, China). HEK293T cells were cultured in DMEM (Gibco) supplemented with 10% FBS. All cell lines were authenticated using short tandem repeat profiling and tested for mycoplasma contamination.

For the methyltransferase inhibitor 5-aza-2′-deoxycytidine (DAC; Sigma-Aldrich, MO, USA) treatment, transfected cells grown for 24 h were treated with or without 10 mM DAC; DAC was re-added every 24 h for 72 h. For proteasome inhibitor MG132 (Selleck, TX, USA) and lysosome inhibitor chloroquine (CQ, Sigma) treatment, transfected cells were incubated in medium containing 10 μM MG132 or 50 μM CQ for 8 h. For protein synthesis inhibitor cycloheximide (CHX, Sigma) treatment, transfected cells were treated with a concentration of 100 μg/mL CHX for the designated time (0, 1, 2, 4, 8 h).

### Plasmid construction and transfection

The DNAJA4, MYH9 and PSMD2 coding sequences (CDSs) were separately tagged with HA, FLAG or Myc and then inserted into empty plasmids to construct the overexpression plasmids pSin-EF2-puro-DNAJA4-HA, pEnter-kana-MYH9-FLAG, pSin-EF2-puro-PSMD2-Myc and pHAGE-CMV-PSMD2-FLAG. The domain deletion mutants DNAJA4-ΔDnaJ, DNAJA4-ΔZinc and PSMD2-ΔPfam were constructed. The shRNA sequences #1 and #2 targeting DNAJA4 and PSMD2 were obtained via the Invitrogen Block-iT RNAi Designer. The shRNA sequences were synthesized and then inserted into the pLKO.1-RFP (Addgene, MA, USA) plasmid to gain pLKO.1-shDNAJA4 #1/2 and shPSMD2 #1/2 plasmids. The overexpress sequences and shRNA sequences are listed in Supplementary Table S1.

For transient transfection, HONE1 or SUNE1 cells were transfected with overexpression or shRNA plasmids using NEOFECT^TM^ DNA transfection reagent (Neofect, Beijing, China) according to the manufacturer’s instructions and harvested after 24 ~ 48 h for further experiments. For stable transfection, the lentiviral packaging plasmids pSPAX2 and pMD2G (Addgene) were co-transfected with the DNAJA4 overexpression or empty plasmid into 293 T cells to prepare the virus-containing supernatant. NPC cells were infected with the virus-containing supernatant, and screened with puromycin (0.5–1 μg/ml; Invitrogen, NY, USA) 48 h post-infection. The transfection efficiency was determined by RT-qPCR and western blot analyses.

### RT-qPCR analysis

TRIzol reagent (Invitrogen) was used to extract total RNA. Reverse transcription was performed using Random Primer and M-MLV Reverse Transcriptase (Promega, Madison, WI, USA) according to the manufacturer’s procedures. Real-time quantitative PCR (RT-qPCR) was performed using SYBR Green PCR Master Mix (Applied Biosystems; Thermo Fisher Scientific, MA, USA) on the CFX96 Real-Time System (Bio-Rad, CA, USA). GAPDH was used as an endogenous control. All the primers are listed in Supplementary Table S1.

### Western blot analysis

Total protein from freshly frozen samples was obtained by crushing the tissues under liquid nitrogen followed by lysing on ice. Total protein from cells was obtained by lysing the cells by sonication and centrifugation at 4 °C. For western blotting, equal amounts (20–50 μg) of proteins were separated by SDS-PAGE and then transferred onto PVDF membranes (Merck Millipore, MA, USA). After blocking with 5% BSA solution, the corresponding membranes were incubated with anti-DNAJA4 (1:1000, ab185553; Abcam, MA, USA), anti-MYH9 (1:1000, 11128-1-AP; Proteintech, Wuhan, China), anti-PSMD2 (1:1000, 14748-1-AP; Proteintech), anti-E-Cadherin (1:1000, 3195 S; Cell Signaling Technology, MA, USA), anti-Vimentin (1:1000, 5741 S; Cell Signaling Technology), anti-Snail (1:1000, 3879 S; Cell Signaling Technology), anti-Slug (1:1000, 9585 S; Cell Signaling Technology) or anti-α-Tubulin (1:5000, 66031-1-AP; Proteintech) primary antibody overnight at 4 °C, and were subsequently incubated with goat anti-mouse or anti-rabbit secondary antibodies (Cell Signaling Technology) at room temperature for 1 h. The protein bands were detected using the enhanced chemiluminescence system.

### Cell viability and clonogenic assays

For the cell viability assay, 800 HONE1 or 1000 SUNE1 cells were plated into 96-well plates. At the indicated time points (0, 1, 2, 3, 4, 5 d), 10 μl of Cell Counting Kit-8 (CCK-8) reagent (TargetMol, MA, USA) per well was added to the 96-well plates for incubation at 37 °C for 2 h. The absorbance at 450 nm was measured by a spectrophotometer.

For the clonogenic assay, 400 HONE1 or 600 SUNE1 cells were suspended and seeded into 6-well plates for attachment. After approximately 7–10 days of culture for colony formation, the plates were washed, and the colonies were fixed using methanol and stained using haematoxylin. Colonies containing more than 50 cells were counted for data analysis.

### Wound healing, transwell migration and invasion assays

For the wound healing assay, 5 × 10^5^ cells were seeded in 6-well plates and cultured in medium supplemented with 10% FBS for approximately 24 h to 90% confluence. Then, a scratch was made in a straight line along the diameter of the well using a 1–10 μl pipette tip, and the medium was replaced with serum-free medium. Images of the wound healing process were captured at 0 h and 24 h using an inverted microscope.

For the migration and invasion assays, Transwell chambers (Corning, NY, USA) equipped with 8.0 μm pore inserts with or without a Matrigel coating (BD Biosciences, CA, USA) were employed. Briefly, transfected cells (0.5 × 10^5^ or 1 × 10^5^) were resuspended in serum-free medium and seeded in the upper cell chamber, while medium supplemented with 10% FBS was added to the lower cell chamber. After 12–16 h of incubation, the cells in the chambers were fixed with methanol and stained with haematoxylin, and the migrated or invaded cells were counted using an inverted microscope (Nikon Eclipse Ti2-U).

### Immunofluorescence staining

Cells were seeded in 24-well plates containing sterile coverslips, cultured to allow attachment and then fixed in 4% paraformaldehyde. After permeabilization using 0.5% Triton X-100 and blocking using 1% BSA-PBS, the fixed cells were incubated with anti-DNAJA4 (1:100, ab185553; Abcam), anti-MYH9 (1:100, 11128-1-AP; Proteintech), anti-PSMD2 (1:100, 14748-1-AP; Proteintech), anti-E-cadherin (1:100, 3195 S; Cell Signaling Technology) or anti-Vimentin (1:100, 5741 S; Cell Signaling Technology) primary antibody overnight at 4 °C. Subsequently, cells were incubated with Alexa Fluor 594- and 488-conjugated IgG secondary antibody (1:1000, A21207 and A21202; Life Technologies, CA, USA), and nuclei were counterstained with Hoechst 33342 dye (Thermo Fisher Scientific). Finally, images were captured with an ultra-high resolution laser confocal microscope (LSM880; Zeiss, Oberkochen, Germany).

### Mass spectrometry and co-immunoprecipitation (co-IP) assay

Cells were lysed, sonicated and centrifuged to collect supernatant as previously described [21–23]. Then, the protein supernatant was incubated with 2.0 μg of an anti-HA-tag antibody (Sigma) or IgG overnight at 4 °C. Subsequently, the mixture was incubated with Pierce Protein A/G Magnetic Beads (Thermo Fisher Scientific). After washing with IP washing buffer, the collected immune complexes were separated by SDS-PAGE and then stained with silver staining. Mass spectrometry (MS) was performed by Wininnovate Bio (Shenzhen, China). The proteins of interest in the coimmunoprecipitate were detected by western blot analysis. The antibodies used were as follows: anti-HA-tag (H6908, Sigma), anti-Flag-tag (F1804, Sigma), anti-DNAJA4 (ab185553; Abcam) and anti-MYH9 (11128-1-AP; Proteintech).

### Animal models

Female BALB/c nude mice (4–5 weeks old; *n* = 40) were obtained from the Animal Facility of the Sun Yat-sen University Cancer Center (Guangzhou, China). For the lymph node metastatic model, 3.0 × 10^5^ SUNE1 cells that stably overexpressed DNAJA4 or the empty plasmid were injected into the foot pad, and the foot tumours were observed every week. After 6 weeks, the mice were sacrificed, and the foot tumours and inguinal lymph nodes on the same side were excised, fixed with 4% formalin, embedded in paraffin and sliced into sections for further experiments.

For the lung metastatic colonization model, 1 × 10^6^ SUNE1 cells that stably overexpressed DNAJA4 or the empty plasmid were injected into the tail vein, and the mice were raised for approximately 8 weeks and then sacrificed. The lung tissues were removed from the thoracic cavities of the mice, fixed, embedded in paraffin and sliced into sections for further experiments. All of the animal experiment protocols were carried out according to the guidelines of the Institutional Animal Care and Use Committee of Sun Yat-sen University Cancer Center (L025501202208008).

### H&E staining and immunohistochemistry

The sections of the foot pad tumours and lung tissues were deparaffinized, rehydrated and stained with haematoxylin and eosin (H&E) for histological examination. The infiltration of cancer cells in the inguinal lymph nodes was visualized with an anti-pan-cytokeratin antibody (ZSGB-Bio, Beijing, China). Immunohistochemistry was performed and evaluated as previously described [21–23]. The sections were incubated with the primary antibody and then labelled with an HRP-conjugated anti-rabbit or anti-mouse secondary antibody. The bound antibodies were visualized with diaminobenzidine, and the sections were counterstained with haematoxylin. All sections were scored by two pathologists according to the immunoreactive scoring (IRS) system.

### Statistical analysis

All statistical analyses were performed using GraphPad Prism version 8.0.1 software. The data are presented as the mean ± SD of three independent experiments. Survival curves were generated using the Kaplan-Meier method and compared using the long-rank test. Multivariate Cox regression model was used to determine independent prognostic factors. Differences between groups were analysed by two-tailed unpaired Student’s *t*-test or the chi-square test. A *p*-value of less than 0.05 was considered to indicate a statistically significant difference.

## Results

### DNAJA4 hypermethylation causes its downregulation in NPC

**Fig. 1:**
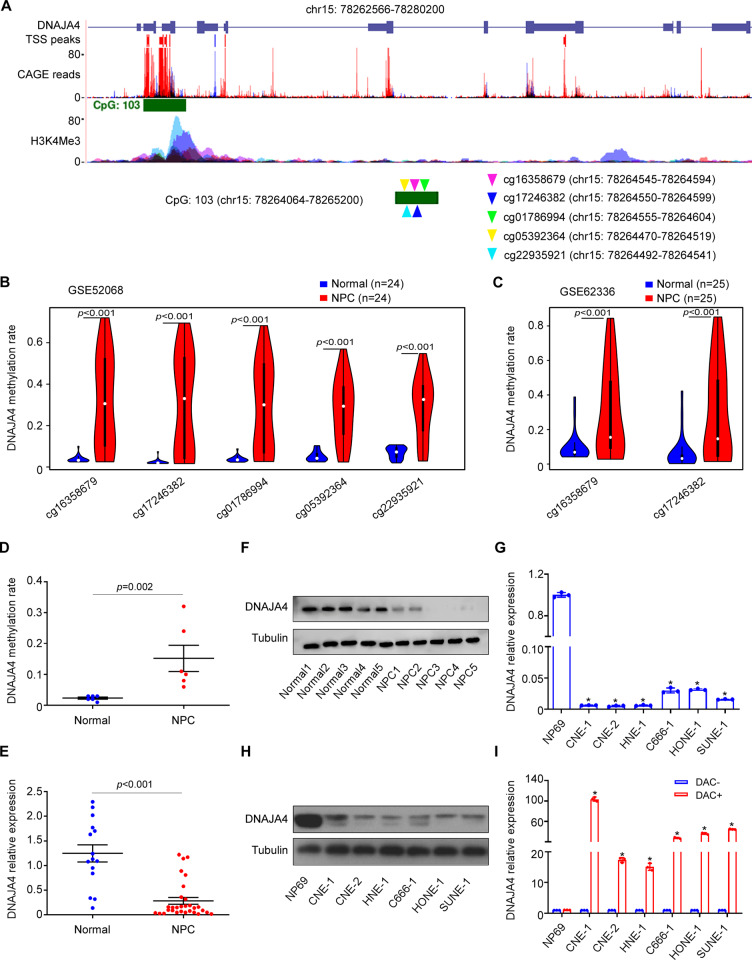
DNAJA4 hypermethylation causes its downregulation in NPC. **A** Features of the DNAJA4 gene observed using the UCSC Genome Browser and a schematic diagram of the CpG island sites in the DNAJA4 promoter region. **B** Methylation levels of DNAJA4 in NPC (*n* = 24) and normal nasopharynx tissues (*n* = 24) in the methylation microarray dataset GSE52068; **C** Methylation levels of DNAJA4 in NPC (*n* = 25) and normal nasopharynx tissues (*n* = 25) in the Hong Kong methylation microarray dataset GSE62336. **D** The methylation rate of DNAJA4 cg16358679 in NPC (*n* = 6) and normal nasopharynx tissues (*n* = 6) based on pyrophosphate sequencing. **E** Relative mRNA levels of DNAJA4 in NPC (*n* = 15) and normal nasopharynx tissues (*n* = 30). **F** Relative protein levels of DNAJA4 in NPC (*n* = 5) and normal nasopharynx tissues (*n* = 5). **G** Relative mRNA levels of DNAJA4 in NPC cell lines and the normal nasopharyngeal epithelial cell line NP69. **H** Relative protein levels of DNAJA4 in NPC cell lines and the normal nasopharyngeal epithelial cell line NP69. **I** Relative mRNA levels of DNAJA4 in NPC cell lines and NP69 cells treated with or without the methyltransferase inhibitor DAC. The data in (**D**, **E**, **G**, **I**) are shown as mean ± SD, and *p*-values were determined by Student’s *t* test (*p < 0.05).

Previous genome-wide DNA methylation microarray data [24] indicated that DNAJA4 was hypermethylated in NPC tissues. Through a search of the UCSG Genome Browser, a CpG island was identified in the DNAJA4 gene. Strong enrichment of H3K4me3, a histone modification that is present at active promoters, was found in the CpG island of DNAJA4 based on ENCODE database analysis, indicating that the CpG island of DNAJA4 might serve as an alternative promoter. Moreover, cap analysis gene expression (CAGE) peaks, which correspond to transcription start sites (TSSs), were also found within the CpG island of DNAJA4 through the FANTOM project (Fig. 1A). In our previous microarray data (GSE52068), five CpG sites of the DNAJA4 gene (cg16358679, cg17246382, cg01786994, cg05392364 and cg22935921), all of which were located within the CpG island, were found to be significantly hypermethylated in NPC tissues compared to normal nasopharynx tissues (Fig. 1B). Analysis of the Hong Kong microarray dataset (GSE62366) confirmed that two of the five CpG sites (cg16358679 and cg17246382) were also markedly hypermethylated in NPC tissues (Fig. 1C).

To verify whether the CpG island of DNAJA4 is hypermethylated in NPC, we measured the methylation level of cg16358679, the most significantly hypermethylated CpG site in the CpG island of DNAJA4, in another 6 NPC and 6 normal nasopharynx tissues by pyrophosphate sequencing, and the results showed that the methylation level of cg16358679 was obviously increased in NPC tissues (Fig. 1D). Next, we measured the mRNA and protein expression levels of DNAJA4 using qRT-PCR and western blotting. The results revealed that both the mRNA and protein expression of DNAJA4 were significantly downregulated in NPC tissues compared to normal nasopharynx tissues (Fig. 1E, F), as well as in NPC cell lines compared to the normal nasopharyngeal epithelial cell line NP69 (Fig. 1G, H). More importantly, we treated NPC cells with the methyltransferase inhibitor DAC and found that the DNAJA4 levels were increased at least 15-fold in the NPC cell lines but not in NP69 cells (Fig. 1I). These findings suggest that the hypermethylation of DNAJA4 causes its downregulation in NPC.

### DNAJA4 suppresses NPC cell migration, invasion and EMT in vitro

**Fig. 2:**
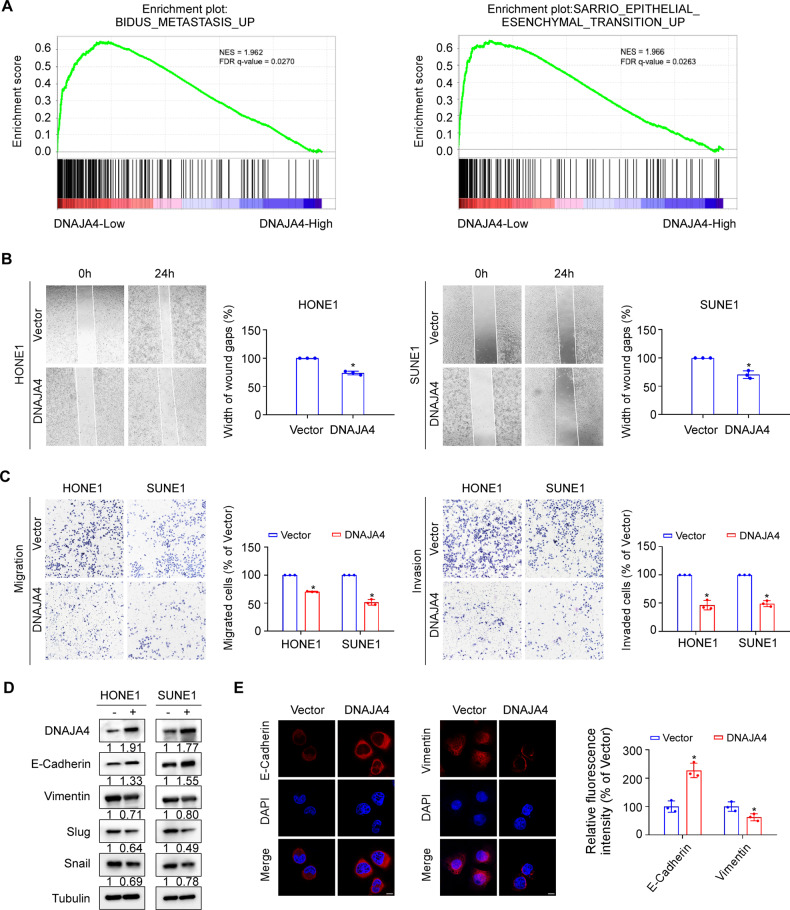
Overexpression of DNAJA4 suppresses NPC cell migration, invasion and EMT. **A** In GSEA of public RNA-seq data (GSE102349), the gene sets related to metastasis and epithelial-mesenchymal transition (EMT) pathways exhibited high enrichment in NPC samples with low DNAJA4 expression. **B** Wound healing assay of HONE1 and SUNE1 cells transfected with the DNAJA4 overexpression or empty plasmid. **C** Transwell migration and invasion assays of HONE1 and SUNE1 cells transfected with the DNAJA4 overexpression or empty plasmid. **D** Representative western blot analysis of the epithelial marker E-cadherin and mesenchymal markers Vimentin, Slug, and Snail in HONE1 and SUNE1 cells transfected with the DNAJA4 overexpression or empty plasmid. **E** Representative immunofluorescence images of the epithelial marker E-cadherin and mesenchymal marker Vimentin in HONE1 cells transfected with the DNAJA4 overexpression or empty plasmids. Scale bar, 50 μm. The data in (**B**, **C**, **E**) are shown as mean ± SD, and *p*-values were determined by Student’s *t* test (*p < 0.05).

To explore the function of DNAJA4 in NPC, we performed GSEA using a public RNA-seq dataset (GSE102349) containing data for 113 NPC samples, and found that the gene sets related to metastasis and epithelial-mesenchymal transition (EMT) pathways exhibited high enrichment in NPC samples with low DNAJA4 expression (Fig. 2A). To further investigate the effect of DNAJA4 on NPC metastasis, we transiently transfected DNAJA4 overexpression plasmids into HONE1 and SUNE1 cells (Supplementary Fig. S1A) and then performed in vitro functional assays. The results of the wound healing assay revealed that overexpression of DNAJA4 markedly inhibited the wound closure ability of NPC cells, demonstrating a decreased migration ability (Fig. 2B). The results of the transwell assays showed that overexpression of DNAJA4 significantly reduced the migratory and invasive abilities of NPC cells (Fig. 2C). However, overexpression of DNAJA4 had no obvious effect on NPC cell viability and proliferation, as determined by CCK8 and colony formation assays (Supplementary Fig. S2). Then, we measured the expression levels of EMT proteins using western blot analysis, and the results showed that overexpression of DNAJA4 increased the level of the epithelial maker E-cadherin while decreasing the level of the mesenchymal markers Vimentin, Slug and Snail (Fig. 2D). Moreover, the results of the immunofluorescence assay confirmed that the epithelial marker E-cadherin was upregulated but the mesenchymal marker Vimentin was downregulated in HONE1 cells transfected with the DNAJA4 overexpression plasmid (Fig. 2E).

**Fig. 3:**
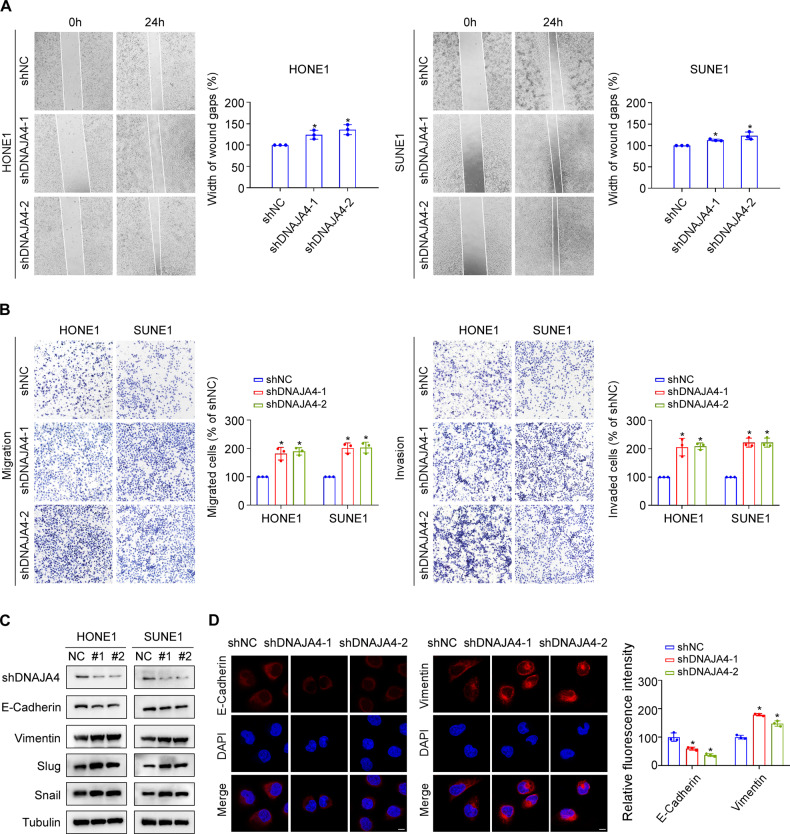
Knockdown of DNAJA4 promotes NPC cell migration, invasion and EMT. **A** Wound healing assay of HONE1 and SUNE1 cells transfected with the shNC or shDNAJA4 plasmid. **B** Transwell migration and invasion assays of HONE1 and SUNE1 cells transfected with the shNC or shDNAJA4 plasmid. **C** Representative western blot analysis of the epithelial marker E-cadherin and mesenchymal markers Vimentin, Slug, and Snail in HONE1 and SUNE1 cells transfected with the shNC or shDNAJA4 plasmid. **D** Representative immunofluorescence images of the epithelial marker E-cadherin and mesenchymal marker Vimentin in HONE1 transfected with the shNC or shDNAJA4 plasmid. Scale bar, 50 μm. The data in (**A**, **B**, **D**) are shown as mean ± SD, and *p*-values were determined by Student’s *t* test (*p < 0.05).

To verify the roles of DNAJA4 in NPC metastasis, we transiently transfected shDNAJA4 plasmids to knock down DNAJA4 expression in HONE1 and SUNE1 cells (Supplementary Fig. S1B). The results of the wound healing and transwell assays revealed that knockdown of DNAJA4 obviously enhanced the migratory and invasive abilities of NPC cells (Fig. 3A, B). Furthermore, the results of the western blot and immunofluorescence staining revealed that knockdown of DNAJA4 increased the levels of the mesenchymal markers Vimentin, Slug and Snail but decreased the levels of the epithelial marker E-cadherin in HONE1 and SUNE1 cells (Fig. 3C, D). Collectively, these results show that DNAJA4 repressed migration and invasion via inactivation of EMT in NPC cells.

### DNAJA4 promotes the degradation of MYH9 by enhancing its ubiquitination

**Fig. 4:**
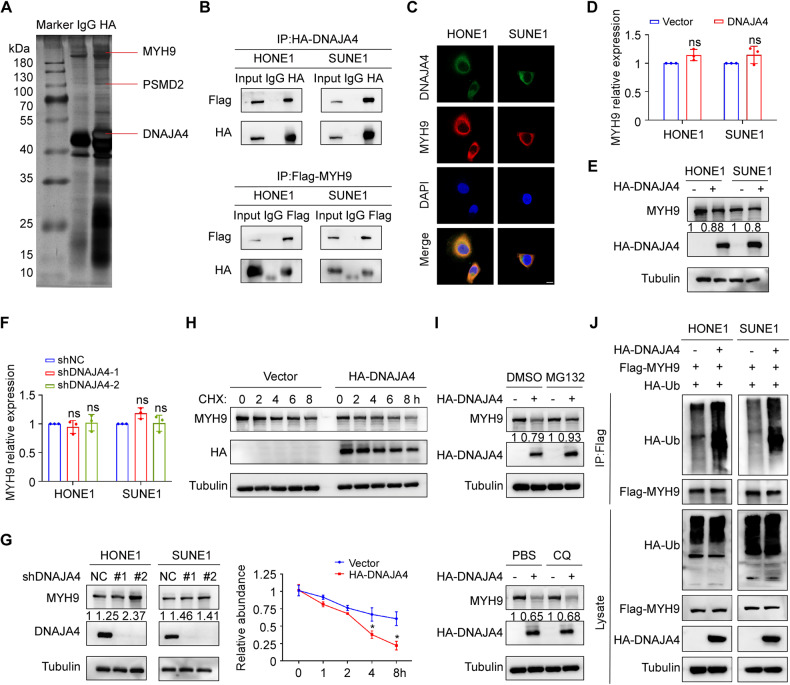
DNAJA4 promotes the degradation of MYH9 by enhancing its ubiquitination. **A** SDS-PAGE and silver staining of proteins isolated from SUNE1 cells transfected with HA-DNAJA4 plasmid and subjected to co-immunoprecipitation with an anti-HA-tag antibody. **B** Co-IP followed by western blotting using anti-HA or anti-Flag antibodies revealed the exogenous association of DNAJA4 and MYH9 in HONE1 and SUNE1 cells. **C** Immunofluorescence staining indicated the cellular localization of endogenous MYH9 and DNAJA4 in HONE1 and SUNE1 cells. Scale bar, 50 μm. **D** The mRNA levels of MYH9 in HONE1 and SUNE1 cells transfected with the DNAJA4 overexpression or empty plasmid. **E** The protein levels of MYH9 in HONE1 and SUNE1 cells transfected with the DNAJA4 overexpression or empty plasmid. **F** The mRNA levels of MYH9 in HONE1 and SUNE1 cells transfected with the shNC or shDNAJA4 plasmid. **G** The protein levels of MYH9 in HONE1 and SUNE1 cells transfected with the shNC or shDNAJA4 plasmid. **H** The effect of CHX treatment and greyscale analysis of MYH9 protein levels in HONE1 cells transfected with the DNAJA4 overexpression or empty plasmid. **I** MG132 and CQ treatment in HONE1 cells transfected with the DNAJA4 overexpression or empty plasmid. **J** HONE1 and SUNE1 cells transfected with the indicated plasmids were subjected to denaturing-IP, and the ubiquitination of MYH9 and the indicated proteins was exogenously detected. The data in (**D**, **F**, **H**) are shown as the mean ± SD, and *p*-values were determined by Student’s *t* test (ns, not significant; *p < 0.05).

To further explore the mechanism by which DNAJA4 affects NPC metastasis, we transiently transfected SUNE1 cells with the HA-tagged DNAJA4 overexpression plasmid and then conducted co-IP with an anti-HA antibody or IgG prior to liquid chromatography-tandem MS analysis. We identified MYH9, which had the largest number of peptide ions matched to DNAJA4, as the potential target (Fig. 4A; Supplementary Table S2 and Fig. S3A). The co-IP results confirmed the exogenous interactions between DNAJA4 and MYH9 (Fig. 4B). Immunofluorescence staining verified the endogenous colocalization between DNAJA4 and MYH9 in the cytoplasm of NPC cells (Fig. 4C). Then, qRT-PCR and western blot analyses showed that neither overexpression nor knockdown of DNAJA4 affected the mRNA level of MYH9, but overexpression of DNAJA4 decreased the protein level of MYH9, and knockdown of DNAJA4 increased the MYH9 level (Fig. 4D–G). In addition, we treated HONE1 cells with CHX to block protein synthesis and then tested the stability of the MYH9 protein. The results demonstrated that overexpression of DNAJA4 greatly facilitated the degradation of endogenous MYH9 protein (Fig. 4H).

To determine whether DNAJA4-mediated MYH9 protein degradation is dependent on the ubiquitin-proteasome or lysosomal pathway, we transfected a DNAJA4 overexpression plasmid or empty plasmid into HONE1 cells and then treated the cells with the proteasome inhibitor MG132 or the lysosome inhibitor CQ. The results showed that DNAJA4-mediated destabilization of MYH9 was reversed by MG132 but not by CQ, indicating that DNAJA4 promotes the degradation of MYH9 in a ubiquitin-proteasome-dependent manner (Fig. 4I). Subsequently, we measured the effects of DNAJA4 on the ubiquitination of MYH9 by denaturing-IP and found that overexpression of DNAJA4 greatly increased the exogenous polyubiquitination of MYH9 (Fig. 4J). Collectively, our data indicate that DNAJA4 promotes the polyubiquitination of MYH9, thus causing its degradation through the ubiquitin-proteasome pathway.

### DNAJA4 recruits PSMD2 to ubiquitinate MYH9 and further causes its degradation

**Fig. 5:**
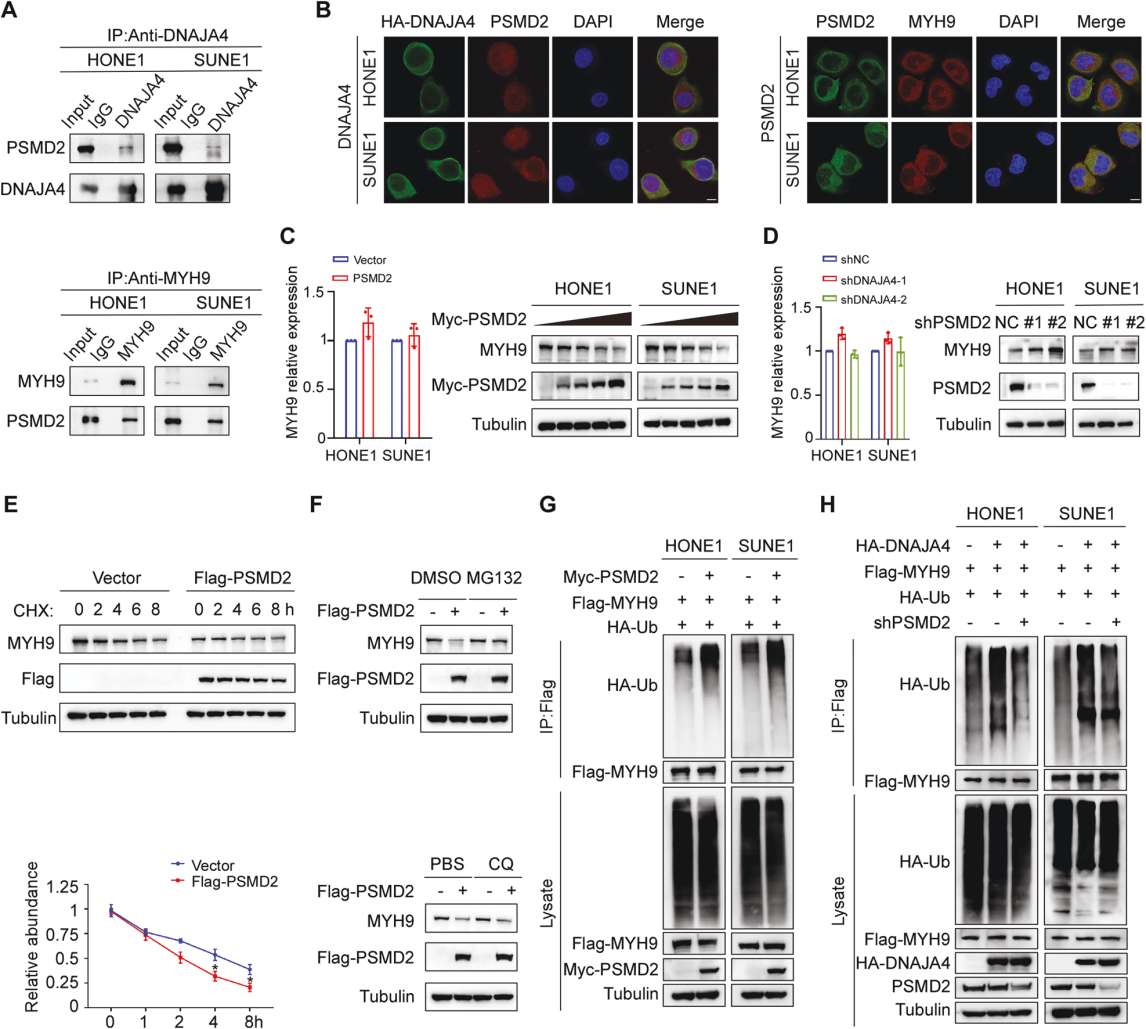
DNAJA4 recruits PSMD2 to ubiquitinate MYH9 and further causes its degradation. **A** Co-IP followed by western blotting using anti-DNAJA4 or anti-MYH9 antibodies revealed the endogenous association of DNAJA4 and PSMD2, as well as MYH9 and PSMD2, in HONE1 and SUNE1 cells. **B** Immunofluorescence staining indicated the cellular localization of exogenous HA-DNAJA4 and endogenous PSMD2 in HONE1 and SUNE1 cells transfected with the HA-DNAJA4 plasmid, as well as the cellular localization of endogenous PSMD2 and MYH9. Scale bar, 50 μm. **C** The mRNA and protein levels of MYH9 in HONE1 and SUNE1 cells transfected with the PSMD2 overexpression or empty plasmid. **D** The mRNA and protein levels of MYH9 in HONE1 and SUNE1 cells transfected with the shNC or shPSMD2 plasmid. **E** The effect of CHX treatment and greyscale analysis of MYH9 protein levels in HONE1 cells transfected with the PSMD2 overexpression or empty plasmid. **F** MG132 and CQ treatment in HONE1 cells transfected with the PSMD2 overexpression or empty plasmid. **G** HONE1 and SUNE1 cells transfected with the indicated plasmids were subjected to denaturing-IP and the ubiquitination of MYH9 and the indicated proteins were exogenously detected. **H** HONE1 and SUNE1 cells transfected with the indicated plasmids or shRNA were subjected to denaturing-IP, and the ubiquitination of MYH9 and the indicated proteins was exogenously detected. The data in (**C**, **D**, **E**) are shown as the mean ± SD, and *p*-values were determined by Student’s *t* test (ns, not significant; *p < 0.05).

The ubiquitin proteasome system has two stages. The first stage constitutes the interaction of the protein substrate with ubiquitin and E1, E2 and E3 enzymes; in the second stage, the protein substrate modified with ubiquitin can be degraded by the proteasome [25]. Through screening and analysis of the MS data, we chose PSMD2, a multicatalytic proteinase complex, for further exploration (Fig. 4A; Supplementary Table S2 and Fig. S3B). The results of co-immunoprecipitation confirmed that PSMD2 cound endogenously bind to not only the DNAJA4 protein, but also the MYH9 protein (Fig. 5A). We then constructed domain deletion mutants of DNAJA4 and PSMD2 and found that the DnaJ domain of DNAJA4 and the Pfam domain of PSMD2 were required for the binding of the DNAJA4 and PSMD2 proteins (Supplementary Fig. S4). Immunofluorescence analysis revealed the colocalization of DNAJA4 and endogenous PSMD2, as well as the endogenous colocalization of PSMD2 and MYH9 in the cytoplasm of both HONE1 and SUNE1 cells (Fig. 5B). Then, we discovered that overexpression of PSMD2 decreased the protein level of MYH9 in a concentration-dependent manner but did not affect its mRNA level (Fig. 5C). Conversely, knockdown of PSMD2 increased the protein level of MYH9 but did not affect its mRNA level (Fig. 5D). Overexpression of PSMD2 significantly facilitated the degradation of endogenous MYH9 in HONE1 cells treated with CHX (Fig. 5E).

Then, we transfected HONE1 cells with the DNAJA4 overexpression plasmid or the corresponding empty plasmid and then treated the cells with the proteasome inhibitor MG132 or the lysosome inhibitor CQ. The results showed that the PSMD2-mediated destabilization of MYH9 was reversed by MG132 but not by CQ, demonstrating that PSMD2 promotes the protein degradation of MYH9 in a ubiquitin-proteasome-dependent manner (Fig. 5F). Subsequently, we quantitatively evaluated the effects of PSMD2 on the ubiquitination of MYH9 via denaturing-IP and found that overexpression of PSMD2 greatly increased the exogenous polyubiquitination of the MYH9 protein in NPC cells (Fig. 5G). Moreover, we performed denaturing-IP after rescue transfection to test whether the ubiquitination of MYH9 mediated by DNAJA4 is affected by PSMD2, and the results showed that knockdown of PSMD2 reversed the exogenous polyubiquitination of MYH9 mediated by DNAJA4 in NPC cells (Fig. 5H). These results suggested that DNAJA4 promotes the polyubiquitination of MYH9 by recruiting PSMD2 in a ubiquitin-proteasome-dependent manner.

### MYH9 reverses the inhibitory effect of DNAJA4 on NPC cell migration, invasion, and EMT

**Fig. 6:**
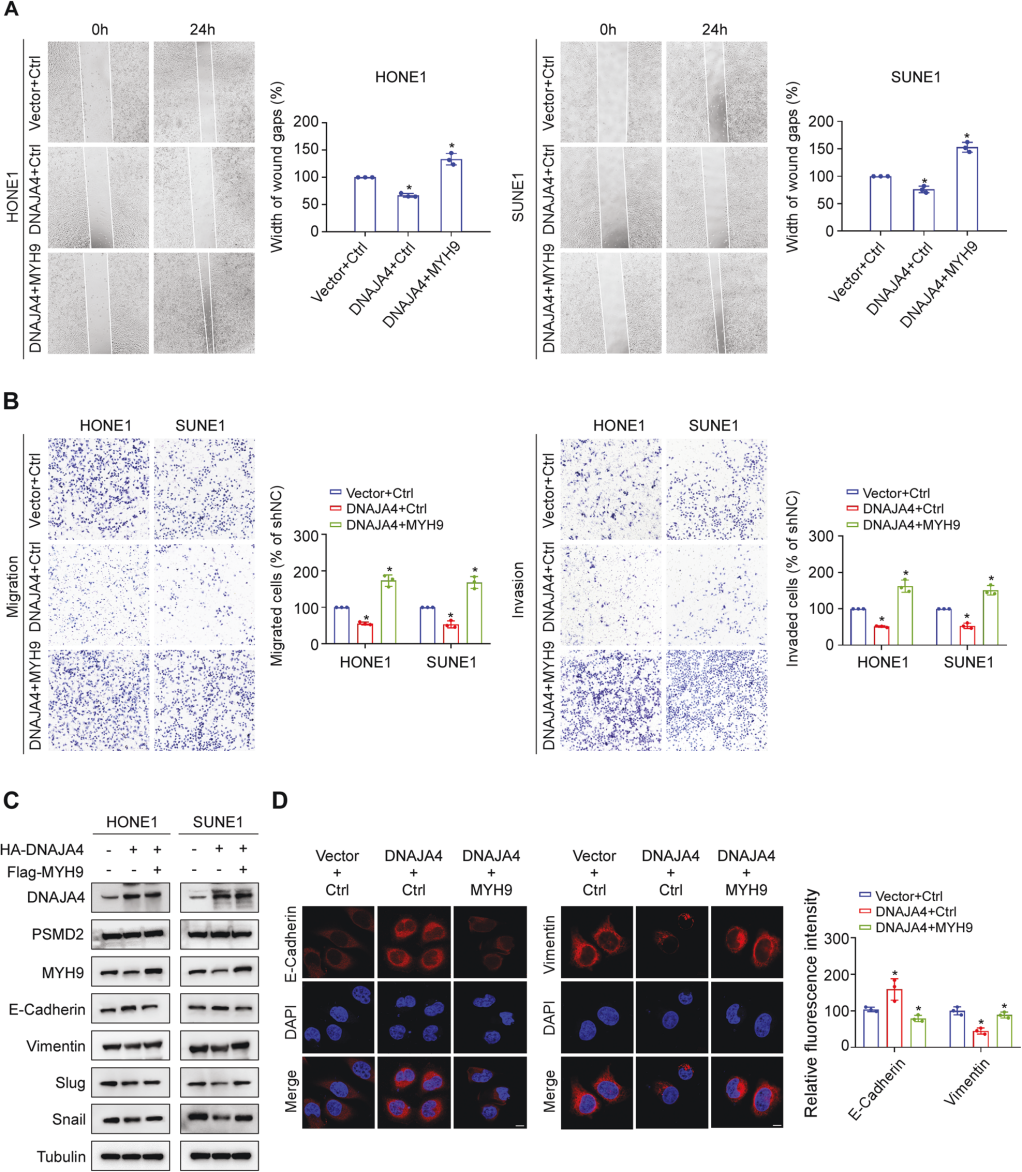
MYH9 reverses the inhibitory effect of DNAJA4 on NPC cell migration, invasion, and EMT. **A** Wound healing assay of HONE1 and SUNE1 cells transfected with the indicated plasmids. **B** Transwell migration and invasion assays of HONE1 and SUNE1 cells transfected with the indicated plasmids. **C** Representative western blot analysis of the epithelial marker E-cadherin and mesenchymal markers Vimentin, Slug, and Snail in HONE1 and SUNE1 cells transfected with the indicated plasmids. **D** Representative immunofluorescence images of the epithelial marker E-cadherin and mesenchymal marker Vimentin in HONE1 transfected with the indicated plasmids. Scale bar, 50 μm. The data in (**A**, **B**, **D**) are shown as the mean ± SD, and *p*-values were determined by Student’s *t* test (*p < 0.05).

To investigate whether DNAJA4 promotes MYH9 protein degradation and thus exerts an inhibitory effect on NPC cell migration, invasion, and EMT, we co-transfected the DNAJA4 overexpression plasmid with the MYH9 overexpression plasmid into HONE1 and SUNE1 cells. The results showed that overexpression of DNAJA4 markedly inhibited NPC cell migration and invasion, which were restored by overexpression of MYH9 (Fig. 6A, B). In addition, we found that overexpression of MYH9 obviously decreased the epithelial marker E-cadherin expression and remarkably increased the mesenchymal markers Vimentin, Slug, and Snail expression, while knockdown of MYH9 increased E-cadherin expression and decreased Vimentin, Slug, and Snail expression in NPC cells (Supplementary Fig. S5). Consistently, the increase in the protein level of E-cadherin as well as the decreases in the protein levels of Vimentin, Snail and Slug induced by DNAJA4 overexpression were reversed by overexpression of MYH9 (Fig. 6C). Similarly, the results of immunofluorescence assay revealed that the fluorescence intensity of Vimentin was decreased but that of E-cadherin was increased in HONE1 cells overexpressing of DNAJA4, and these changes were also reversed by overexpression of MYH9 (Fig. 6D). These results demonstrate that MYH9 can reverse the inhibitory effects of DNAJA4 on NPC cell migration, invasion, and EMT.

### DNAJA4 inhibits NPC cell invasion and metastasis in vivo

**Fig. 7:**
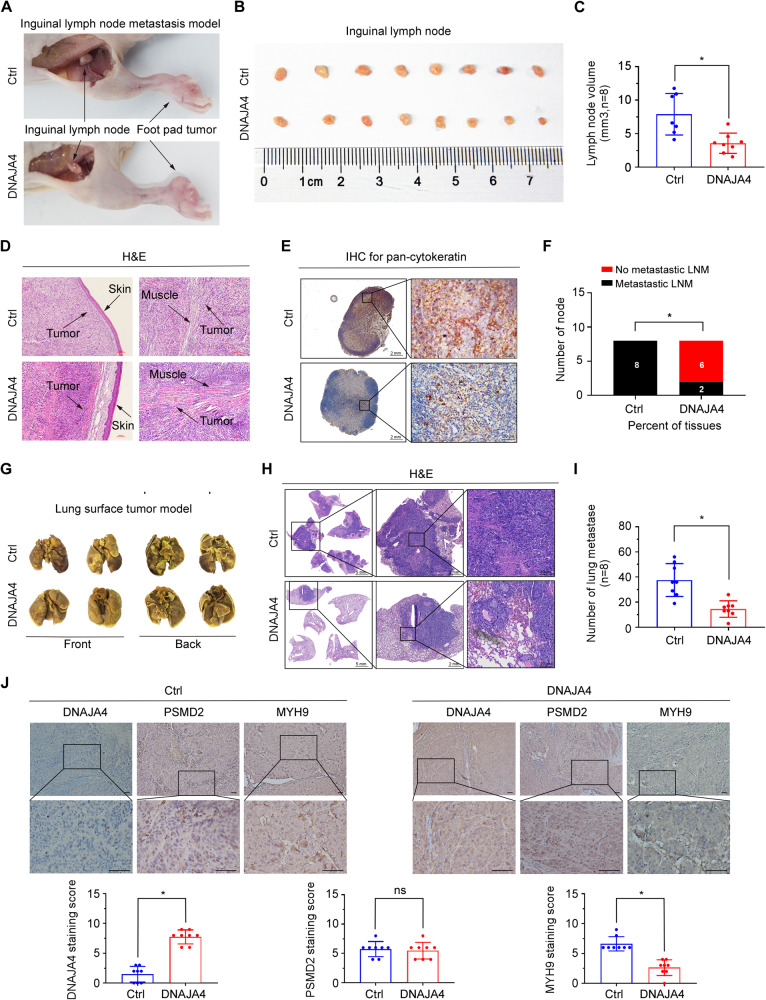
DNAJA4 inhibits NPC invasion and metastasis in vivo. SUNE1 cells stably overexpressing DNAJA4 or empty vector were subcutaneously injected into the plantar surface of mice to establish the inguinal lymph node metastasis model. **A**, **B** Representative images of foot pad tumours (**A**) and inguinal lymph nodes (**B**) in the two groups. **C** Statistical comparison of lymph node volume between the two groups. **D** H&E staining of foot pad tumours to analyse the infiltration of cancer cells into mouse muscle and skin. Scale bar, 100 μm. **E** The infiltration of cancer cells into lymph nodes was evaluated using IHC analysis of pan-cytokeratin. Scale bars, 2 mm and 20 μm. **F** Statistical analysis of inguinal lymph node metastasis in the two groups. SUNE1 cells stably overexpressing DNAJA4 or empty vector were injected into the tail veins of mice to establish the lung metastatic colonization model. **G** Representative images of macroscopic tumour nodules formed on the lung surface in the two groups. **H** H&E staining of the lung tissues to analyse the number and size of metastatic nodules. Scale bars, 5 mm, 2 mm and 20 μm. **I** Statistical analysis of lung metastatic nodules in the two groups. **J** Representative images and quantitative analysis of IHC staining of DNAJA4, PSMD2, and MYH9 in transplanted tumour tissues of the two groups. Scale bars, 100 μm and 50 μm. The data in (**C**, **I**, **J**) are shown as the mean ± SD, and *p*-values were determined by Student’s *t* test (ns, not significant; *p < 0.05).

To identify whether DNAJA4 facilitates the metastasis of NPC cells in vivo, we established inguinal lymph node metastasis and lung metastatic colonization models. The results showed that the inguinal lymph nodes in the DNAJA4-overexpression group displayed smaller sizes and volumes than those in the control (Ctrl) group (Fig. 7A–C). H&E staining of the foot pad tumours demonstrated that the tumours in the DNAJA4-overexpression group exhibited less aggressive behaviour, characterized by intrusive invasions into the skin or muscle, than those in the Ctrl group (Fig. 7D). The results of IHC staining of pan-cytokeratin in inguinal lymph nodes also suggested that the DNAJA4-overexpression group had markedly fewer metastases than the Ctrl group (Fig. 7E, F). In addition, H&E staining of lung tissues revealed that the DNAJA4-overexpression group presented fewer and smaller nodules on the lung surface (Fig. 7G) and in lung slices (Fig. 7H, I) than did the Ctrl group. The results of IHC assay revealed that MYH9 was downregulated in DNAJA4-overexpressing tumours, while PSMD2 expression had no obvious change (Fig. 7J). The observations in these metastasis models verify that DNAJA4 suppresses the metastasis of NPC cells in vivo.

### Low expression of DNAJA4 indicates poor prognosis in NPC patients

**Fig. 8:**
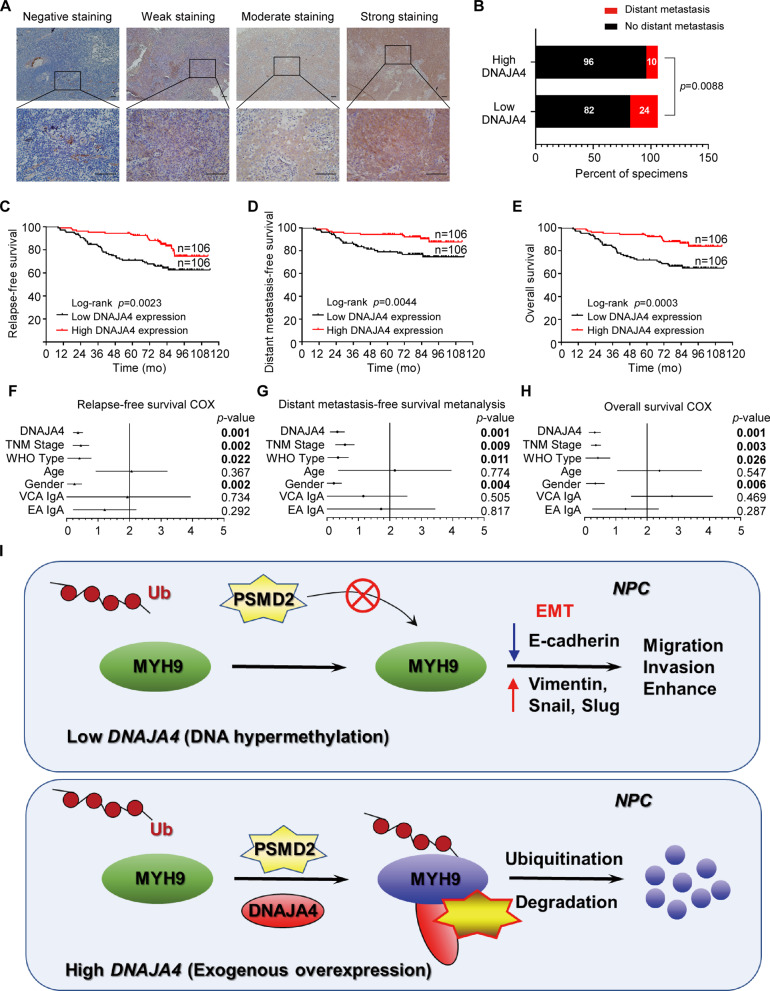
Low expression of DNAJA4 indicates poor prognosis in NPC patients. **A** Representative images of IHC staining of DNAJA4 in 212 NPC tissues. The staining of the images was graded as negative, weak, moderate, or strong based on the staining intensity. Scale bars, 100 μm and 20 mm. **B** Correlations of distant metastasis status with the level of DNAJA4 determined by IHC staining. The p-values were calculated using the chi-square test. **C**–**E** Kaplan-Meier curves of relapse-free survival (**C**), distant metastasis-free survival (**D**) and overall survival (**E**) based on DNAJA4 protein level. The p-values were determined using the log-rank test. **F**–**H** Forest plots of multivariate Cox regression analyses showing the significance of different clinical prognostic factors for NPC relapse-free survival (**F**), distant metastasis-free survival (**G**) and overall survival (**H**). **I** The regulatory model of DNAJA4 in NPC. When promoter hypermethylation of DNAJA4 causes its downregulation in NPC cells, the expression of MYH9 is not inhibited, and MYH9 then facilitates the migration and invasion of NPC cells by activating EMT. After exogenous overexpression of DNAJA4, DNAJA4 recruits PSMD2 to promote the protein degradation of MYH9 in a ubiquitination-dependent manner, and finally, the migration and invasion mediated by EMT are blocked.

To further explore the clinical significance of DNAJA4 in NPC patients, we measured the protein levels of DNAJA4 in 212 NPC tissues using IHC staining. We detected positive staining of DNAJA4 in the cytoplasm of cells in NPC tissues, and found different expression levels of DNAJA4 (negative, weak, moderate or strong) according to the staining intensity (Fig. 8A). The results indicated that NPC patients with low DNAJA4 expression were more prone to distant metastasis (Fig. 8B). Based on the IHC scores of DNAJA4 in NPC tissues, we divided these patients into the low DNAJA expression group and the high DNAJA4 expression group for Kaplan-Meier analysis, and the survival curves demonstrated that the low DNAJA4 expression group had lower relapse-free, distant metastasis-free and overall survival rates than the high DNAJA4 expression group (Fig. 8C–E). Cox regression analysis further showed that DNAJA4 expression and TNM stage were independent prognostic indicators for NPC **(**Fig. 8F–H). In summary, these results demonstrate that low expression of DNAJA4 indicates a poor prognosis and is correlated with tumour metastasis in NPC patients.

## Discussion

In this study, we revealed that the promoter hypermethylation of DNAJA4 caused its downregulation in NPC. Overexpression of DNAJA4 significantly suppressed NPC cell migration, invasion, and EMT in vitro, and markedly inhibited inguinal lymph node metastasis and lung metastatic colonization in vivo, while it did not affect NPC cell viability and proliferation capability. Mechanistically, DNAJA4 facilitated the protein degradation of MYH9 via the ubiquitin-proteasome pathway by recruiting PSMD2. Furthermore, the suppressive effects of DNAJA4 on NPC cell migration, invasion, and EMT were reversed by overexpression of MYH9 in NPC cells. Clinically, a low level of DNAJA4 expression indicated poor prognosis and an increased probability of distant metastasis in NPC patients.

Metastasis is the main cause of death and treatment failure in NPC patients, and great efforts have been devoted to discovering the underlying molecular mechanism involved in the invasion and metastasis of NPC in recent decades [26–30]. Aberrant DNA methylation is regarded as a common and key event in human cancers, and the reversible nature of the DNA methylation process makes it a potential target for cancer treatment [31, 32]. Compared with genetic changes such as mutation and copy number variation, DNA methylation is a more important molecular event in the tumorigenesis and progression of NPC [33, 34]. Dai et al. reported that more than 90% of the differentially methylated CpG sites were hypermethylated in NPC tissues [35], suggesting that the epigenetic silencing of functional genes may play vital roles in NPC pathology. Many tumour suppressor genes have been demonstrated to be inactivated in NPC because of promoter hypermethylation [21, 22, 36–39]. Here, based on genome-wide DNA methylation profiling, we revealed that the DNAJA4, a member of the HSP40/DNAJ family, was hypermethylated, causing its downregulation in NPC. Promoter hypermethylation of DNAJA4 has also been reported in several other diseases, such as paediatric embryonal and alveolar rhabdomyosarcomas, Ewing sarcoma, and oral leukoplakia [40–42]. Subsequently, in vitro and in vivo functional experiments showed that overexpression of DNAJA4 suppressed NPC cell migration, invasion, EMT and metastasis. More importantly, patients with low expression of DNAJA4 had a poor prognosis and were proven to be easily develop distant metastasis. Our findings add evidence that NPC is a disease featuring abnormal methylation.

Subsequently, we performed MS analysis and identified MYH9 as a regulatory target of DNAJA4. DNAJA4 promoted the protein degradation of MYH9, but it did not affect its mRNA level. MYH9, also called non-muscle myosin heavy chain IIA, belongs to the myosin II subfamily [43]. MYH9 is involved in the generation of cell polarity, cell-cell adhesion processes and maintenance of the cytoskeletal structure by binding to actin filaments [44], and is also involved in invasion and metastasis in human cancers [45]. MYH9 can promote cancer cell EMT and metastasis by regulating multiple signal transduction pathways. For instance, MYH9 facilitates the cell EMT and migration of colorectal cancer by activating MAPK/AKT signalling [46], and promotes cell invasion and radioresistance in head and neck cancer by modulating the cellular ROS levels via activation of the MAPK/Nrf2/GCLC axis [47]. Human tubulin beta class IVa (TUBB4A) interacts with MYH9 to protect against nuclear DNA damage and promotes cell EMT and migration via activation of GSK3β/β-catenin signalling in prostate cancer [48]. Sterile alpha motif domain-containing protein 9 (SAMD9) promotes EMT and metastasis of esophageal squamous cell carcinoma by stimulating MYH9-mediated GSK3β/β-catenin signalling [49]. In addition, S100A4 interacts with MYH9 to promote the migration and invasion of gastric cancer cells via TGF-β-mediated EMT [50]. MYH9 increases the proliferative and metastatic potential of renal cell carcinoma cells by stimulating AKT signalling pathway [51]. CRLF1 interacts with MYH9 to promote cell proliferation and metastasis via the ERK/ETV4 axis in papillary thyroid carcinoma [52]. Here, we reveal that DNAJA4 interacts with MYH9 to promote its degradation, thus promoting cell migration and invasion via EMT in NPC.

Recently, increasing evidence has shown that the protein level of MYH9 can be regulated by the ubiquitin-proteasome pathway. CircRNA EIF6-224aa directly interacts with the MYH9 protein and impedes MYH9 degradation by suppressing the ubiquitin-proteasome pathway, subsequently activating the Wnt/beta-catenin signalling pathway in triple-negative breast cancer [53]. MAP7D2 interacts with the MYH9 protein to protect it from ubiquitin-mediated degradation, subsequently reducing the secretion of HMGB1 to inhibit the infiltration of CD8^+^ cytotoxic T lymphocytes in microsatellite-stable colorectal cancer [54]. Conversely, the lncRNA TPRG1-AS1 directly interacts with MYH9 to promote its degradation through the proteasome pathway, subsequently hindering F-actin stress fiber formation and finally inhibiting the migration of human aortic smooth muscle cells [55]. In this study, we found that the proteasomal noncatalytic subunit PSMD2 interacted with MYH9 and induced its degradation via the ubiquitin-proteasome pathway in NPC. PSMD2, proteasome 26 S subunit ubiquitin receptor/ non-ATPase 2, is reported to be a key ubiquitin-proteasome-related gene for predicting prognosis in multiple cancer types [56–58]. PSMD2 physically interacts with p21 and p27 and mediates their degradation via the ubiquitin-proteasome pathway by cooperating with the deubiquitinating enzyme USP14, subsequently promoting cell proliferation and facilitating cell cycle progression in breast cancer [59]. PSMD2 also reduces the protein levels of a variety of Ras-related GTPase, including DIRAS family GTPase 2 (DIRAS2) through the proteasome-mediated pathway and subsequently blocks the NF-κB signalling pathway and suppresses cell proliferation in colorectal cancer [60]. In this study, we revealed that DNAJA4 recruited PSMD2 to promote MYH9 protein degradation via the ubiquitin-proteasome pathway and subsequently inhibited the migration and invasion of NPC cells. However, as PSMD2 is not an E3 ligase, the E3 ligase that is recruited by the DNAJA4/PSMD2 complex to promote MYH9 ubiquitination and degradation warrants further investigation.

## Conclusions

In conclusion, DNAJA4 promoted MYH9 protein degradation via the ubiquitin-proteasome pathway by interacting with PSMD2. In vitro, DNAJA4 strongly suppressed cell migration, invasion and EMT, which could be rescued by overexpression of MYH9 in NPC cells. DNAJA4 also strongly inhibited inguinal lymph node metastasis and lung metastasis of NPC cells in vivo (Fig. 8I). Our study demonstrates a novel mechanism of the DNAJA4-PSMD2-MYH9 axis in regulating NPC cell invasion and metastasis and provides new potential targets for NPC.

### Reporting summary

Further information on research design is available in the Nature Research Reporting Summary linked to this article.

### Supplementary information

Supplementary Information

Reporting Summary

## Data Availability

The key raw data generated in this study have been deposited into the Research Data Deposit platform (www.researchdata.org.cn), with the approval RDD number as RDDB2023184613.
