# Supplementary material for: DNAJA4 suppresses epithelial-mesenchymal transition and metastasis in nasopharyngeal carcinoma via PSMD2-mediated MYH9 degradation
Source: Cell Death Dis. 2023 Oct 24;14(10):697. doi: 10.1038/s41419-023-06225-w (PMC10598267; doi:10.1038/s41419-023-06225-w)
Supplement: Supplementary file 1 — Supplementary Information [file 41419_2023_6225_MOESM1_ESM.docx]

**Supplementary Information**

**DNAJA4 suppresses epithelial-mesenchymal transition and metastasis in nasopharyngeal carcinoma via PSMD2-mediated MYH9 degradation**

Qun Zhang^1,*^, Ping Feng,^2,*^ Xun-Hua Zhu^2,*^, Shi-Qing Zhou^2,*^, Ming-Liang Ye^2^, Xiao-Jing Yang^2^, Sha Gong^2^, Sheng-Yan Huang^2^, Xi-Rong Tan^2^, Shi-Wei He^2,†^, Ying-Qing Li^2,†^


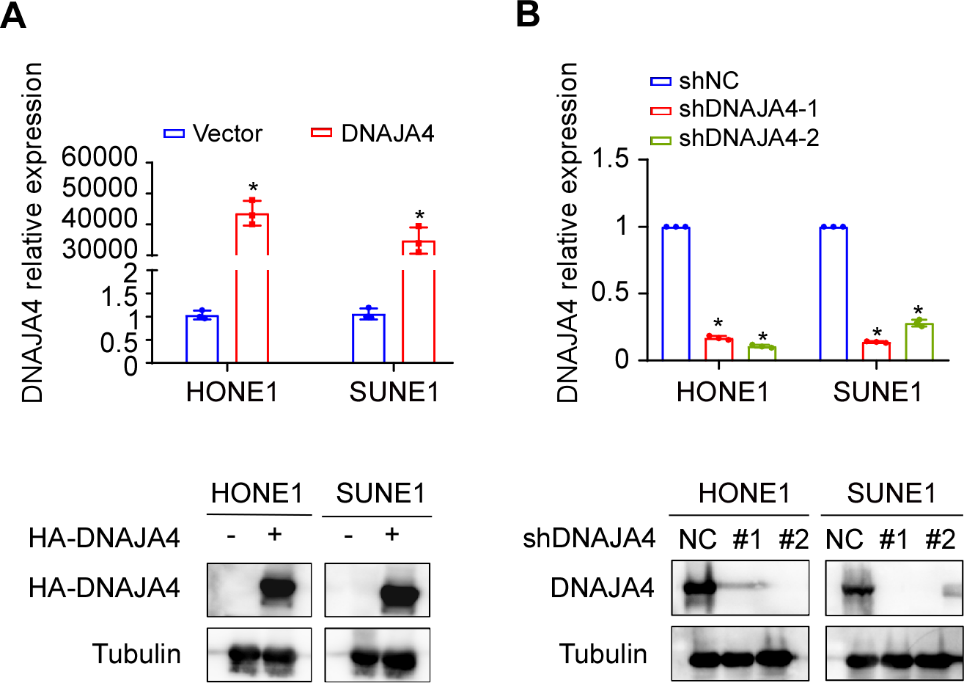


**Supplementary Fig. 1 Relative levels of DNAJA4 after transient transfection**

A Relative DNAJA4 mRNA and protein levels in HONE1 and SUNE1 cells after transient transfection with the DNAJA4 overexpression or empty plasmid. B Relative DNAJA4 mRNA and protein levels in HONE1 and SUNE1 cells after transient transfection with the shNC or shDNAJA4 plasmid. The data are shown as the mean±SD, and the *p*-values were determined by Student’s *t* test (*p < 0.05).


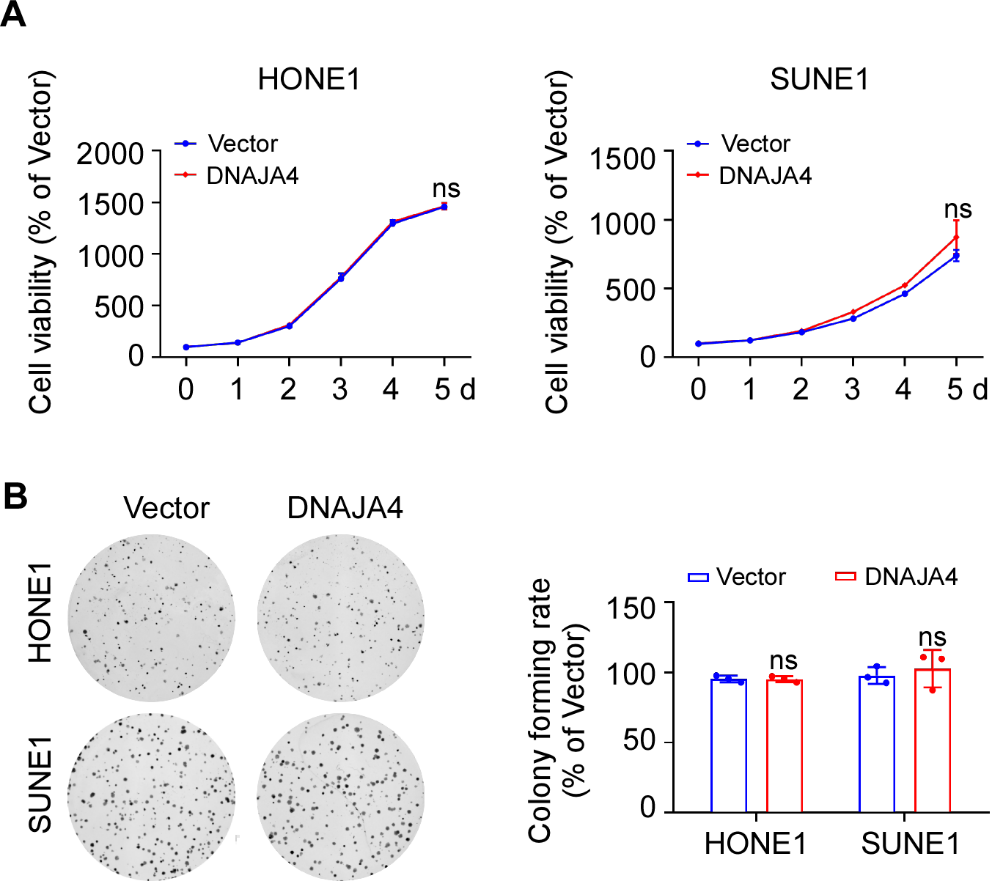


**Supplementary Fig. 2 Overexpression of DNAJA4 has no effect on NPC cell proliferation**

**A** CCK-8 assay was performed to measure the viability of HONE1 and SUNE1 cells transfected with the DNAJA4 overexpression or empty plasmid. **B** A colony formation assay was conducted to assess the colony formation of HONE1 and SUNE1 cells transfected with the DNAJA4 overexpression or empty plasmid. The data are shown as mean±SD, and *p*-values were determined by Student’s *t* test (ns, no significant).


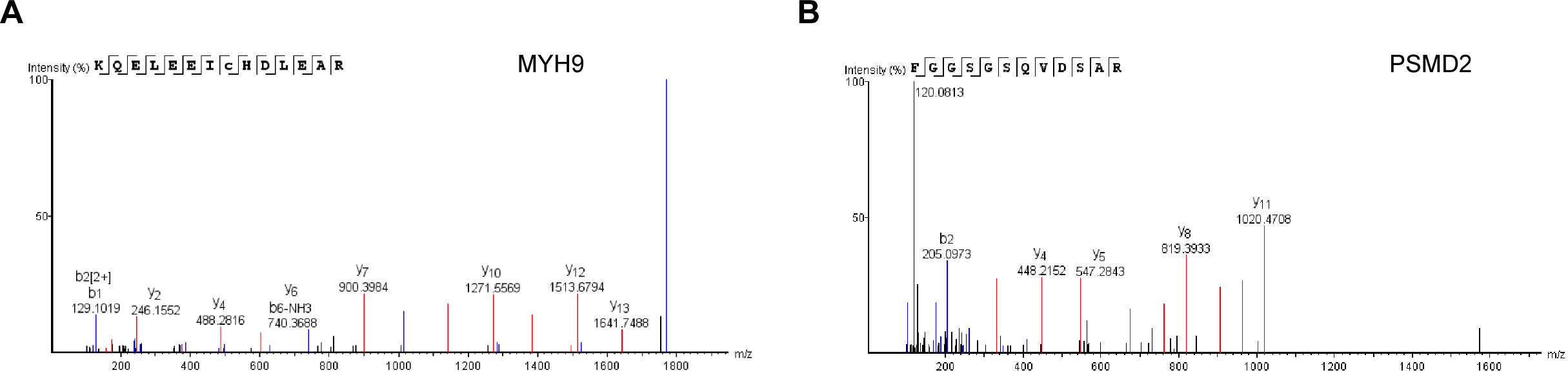


**Supplementary Fig. 3 DNAJA4 junction-specific peptides detected by mass spectrometry**

Specific peptides of MYH9 (**A**) and PSMD2 (**B**) were identified by immunoprecipitation with an anti-HA antibody in SUNE1 cells transfected with the HA-DNAJA4 plasmid.


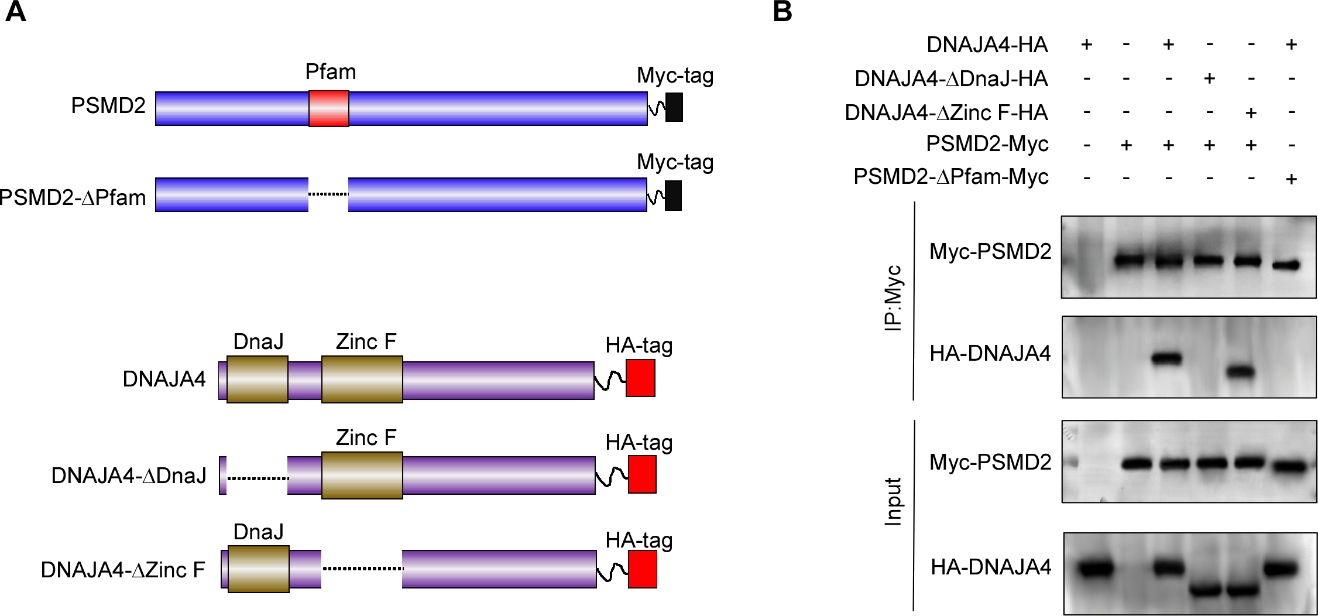


**Supplementary Fig. 4 The interaction relationship between PSMD2 and DNAJA4**

(**A**) Schematic diagram of the PSMD2 and DNAJA4 domain-deletion mutants. (**B**) Co-IP followed by western blotting using an anti-Myc antibody revealed the interactions of HA-DNAJA4 or the HA-tagged DNAJA4 domain deletion mutants with Myc-PSMD2 or the Myc-tagged PSMD2 domain deletion mutant.


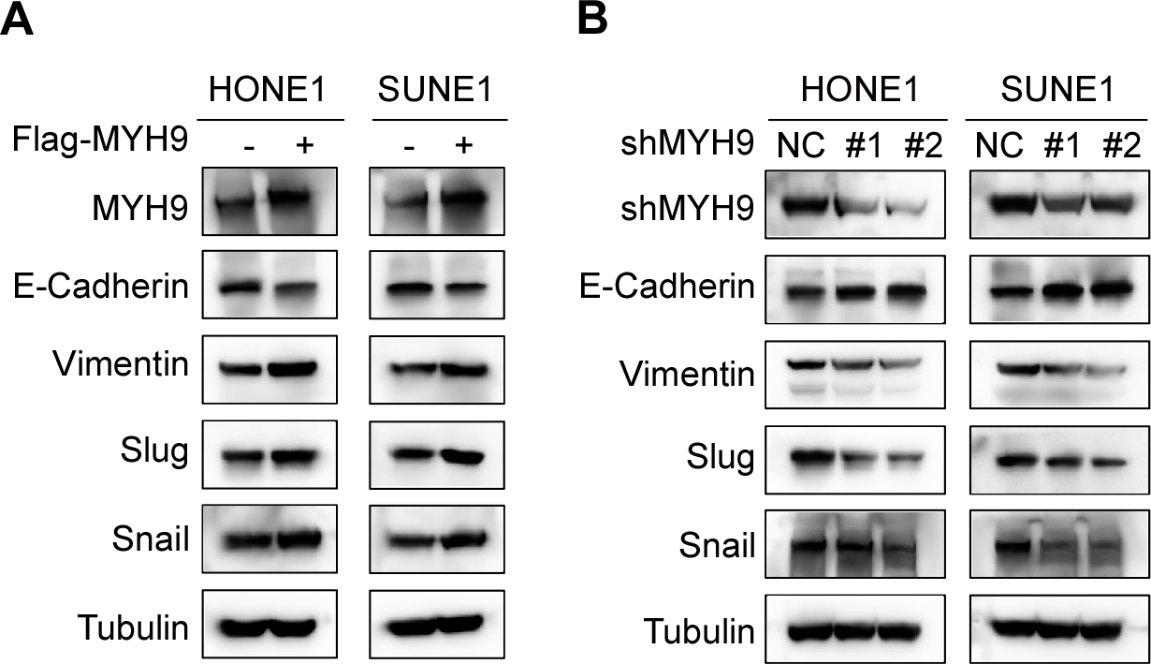


**Supplementary Fig. 5 Overexpression of MYH9 promotes EMT in NPC**

(**A**) Western blot showing that overexpression of MYH9 decreased E-cadherin expression, while increased Vimentin, Slug, and Snail expression in HONE1 and SUNE1 cells. (**B**) Western blot showing that knockdown of MYH9 increased E-cadherin expression, while decreased Vimentin, Slug, and Snail expression in HONE1 and SUNE1 cells.

**Supplementary Table 1 Primers for RT-qPCR, shRNAs and vector constructions**

| **Name** | **Sequences (5'-3')** | **Description** |
| --- | --- | --- |
| **Primers for RT-qPCR from freshly-frozen tissues and cells** | |  |
| DNAJA4 F | GAATGCCCATCTACAAAGCACCC | Homo sapiens DNAJA4 QPCR Primer,  product length = 132 |
| DNAJA4 R | TCTGTCGAGGAGGGAGTAAAGC |  |
| PSMD2 F | TGCTCGTGGAACGACTAGG | Homo sapiens PSMD2 QPCR Primer,  product length = 146 |
| PSMD2 R | CAGTTTGCCATAGTGTGGACG |  |
| MYH9 F | ATCCTGGAGGACCAGAACTGCA | Homo sapiens MYH9 QPCR Primer,  product length = 114 |
| MYH9 R | GGCGAGGCTCTTAGATTTCTCC |  |
| GAPDH F | GTCTCCTCTGACTTCAACAGCG | Homo sapiens GAPDH QPCR Primer,  product length = 131 |
| GAPDH R | ACCACCCTGTTGCTGTAGCCAA |  |
| **shRNA sequences cloned into pLKO.1-RFP plasmid** | |  |
| shDNAJA4-1-AgeI F | CCGGTGCGAGAAGTTTAAACTCATATCTCGAGATATGAGTTTAAACTTCTCGCTTTTTG | |
| shDNAJA4-1-EcoRI R | AATTCAAAAAGCGAGAAGTTTAAACTCATATCTCGAGATATGAGTTTAAACTTCTCGCA | |
| shDNAJA4-2-AgeI F | CCGGTCAGAAGGATCATAGTGTCTTTCTCGAGAAAGACACTATGATCCTTCTGTTTTTG | |
| shDNAJA4-2-EcoRI R | AATTCAAAAACAGAAGGATCATAGTGTCTTTCTCGAGAAAGACACTATGATCCTTCTGA | |
| shPSMD2-1-AgeI F | CCGGTCGCCAGTTAGCTCAATATCATCTCGAGATGATATTGAGCTAACTGGCGTTTTTG | |
| shPSMD2-1-EcoRI R | AATTCAAAAACGCCAGTTAGCTCAATATCATCTCGAGATGATATTGAGCTAACTGGCGA | |
| shPSMD2-2-AgeI F | CCGGTCCACATTTGTAGCGAACACTTCTCGAGAAGTGTTCGCTACAAATGTGGTTTTTG | |
| shPSMD2-2-EcoRI R | AATTCAAAAACCACATTTGTAGCGAACACTTCTCGAGAAGTGTTCGCTACAAATGTGGA | |
| shMYH9-1-AgeI F | CCGGTGACAGCAATCTGTACCGCATTCTCGAGAATGCGGTACAGATTGCTGTCTTTTTG | |
| shMYH9-1-EcoRI R | AATTCAAAAAGACAGCAATCTGTACCGCATTCTCGAGAATGCGGTACAGATTGCTGTCA | |
| shMYH9-2-AgeI F | CCGGTGCCAAGCTCAAGAACAAGCATCTCGAGATGCTTGTTCTTGAGCTTGGCTTTTTG | |
| shMYH9-2-EcoRI R | AATTCAAAAAGCCAAGCTCAAGAACAAGCATCTCGAGATGCTTGTTCTTGAGCTTGGCA | |
| **Primers for plasmid constructs** | | **Cloned into pSin-EF2-puro plasmid** |
| DNAJA4-BamHI F | CGCGGATCCATGGTGAAGGAGACCCAGTACTAT | Construction of DNAJA4 ORF |
| DNAJA4-SpeI-HA R | CGGACTAGTAGCGTAGTCTGGGACGTCGTATGGGTATGCCGTCTGGCACTGCACTCCA | Construction of DNAJA4 ORF with  C-terminal HA tag |
| DNAJA4-ΔDnaJ-BamHI F | CGCGGATCCATGGTGAAGGCAATTAAAGAAGGAGGCTCAGGC | Construction of DNAJA4-ΔDnaJ |
| DNAJA4-ΔDnaJ/DNAJA4-ΔZinc-F-SpeI-HA R | CGGACTAGTAAGCGTAGTCTGGGACGTCGTATGGGTATGCCGTCTGGCACTGCACTCC | Construction of DNAJA4-ΔDnaJ with C-terminal HA tag, DNAJA4-ΔZinc-F with C-terminal HA tag, and DNAJA4 truncation 207-397aa |
| DNAJA4-ΔZinc-F-BamHI F | CGCGGATCCATGGTGAAGGAGACCCAGTAC | Construction of DNAJA4-ΔZinc-F and DNAJA4 truncation 1-121aa |
| DNAJA4-ΔZinc-F-1 F | GAAGATCTATATAATATTATCGAGGTACAT | Construction of DNAJA4 truncation 207-397aa |
| DNAJA4-ΔZinc-F-1 R | ATGTACCTCGATAATATTATATAGATCTTC | Construction of DNAJA4 truncation 1-121aa |
| PSMD2-BamHI F | CGCGGATCCATGGAGGAGGGAGGCCGGGACAAG | Construction of PSMD2 ORF |
| PSMD2-SpeI-Myc R | CGGACTAGTCAGATCCTCTTCAGAGATGAGTTTCTGCTCCATGAGATCATAATTGGGGTTCTTCCG | Construction of PSMD2 ORF with  C-terminal Myc tag |
| PSMD2-ΔPfam-BamHI F | CGCGGATCCATGGAGGAGGGAGGCCGGGAC | Construction of PSMD2-ΔPfam and PSMD2 truncation 1-442aa |
| PSMD2-ΔPfam-SpeI-Myc R | CGGACTAGTCAGATCCTCTTCAGAGATGAGTTTCTGCTCGAGATCATAATTGGGGTTCT | Construction of PSMD2-ΔPfam with C-terminal Myc tag and PSMD2 truncation 590-909aa |
| PSMD2-ΔPfam -1 F | GACTACATTAAGTCAGCCAACACACTGGTG | Construction of PSMD2 truncation 590-909aa |
| PSMD2-ΔPfam -1 R | CACCAGTGTGTTGGCTGACTTAATGTAGTC | Construction of PSMD2 truncation 1-442aa |
| PSMD2-BamHI F | CGCGGATCCATGGAGGAGGGAGGCCGGGACAAG | Construction of PSMD2 ORF |
| PSMD2-Spe-Flag R | CGGACTAGTCTTGTCATCGTCGTCCTTGTAATCGAGATCATAATTGGGGTTCTTCCG | Construction of PSMD2 ORF with  C-terminal Flag tag |
| MYH9-BamHI F | CGCGGATCCATGGCACAGCAAGCTGCCGATAAG | Construction of MYH9 ORF |
| MYH9-SpeI-Flag R | CGGACTAGTCTTGTCATCGTCGTCCTTGTAATCTTCGGCAGGTTTGGCCTCAGC | Construction of MYH9 ORF with  C-terminal Flag tag |

Annotation: *AgeI* restriction site: ACCGGT. *EcoRI* restriction site: GAATTC. *BamHI* restriction site: GGATCC; *SpeI* restriction site: ACTAGT. HA tag sequences: TACCCATACGACGTCCCAGACTACGCT. Myc tag sequences: GAGCAGAAACTCATCTCTGAAGAGGATCTG. Flag tag sequences: GATTACAAGGACGACGATGACAAG. DNAJA4-ΔDnaJ: DNAJA4 Δ4-70, or DNAJA4 truncation 1-3aa linked truncation 71-397aa. DNAJA4-ΔZinc-F: DNAJA4 Δ122-206, or DNAJA4 truncation 1-121aa linked truncation 207-397aa. PSMD2-ΔPfam: PSMD2-Δ443-589, or PSMD2 truncation 1-442aa linked truncation 590-909aa.

| **Supplementary Table 2 Specific interacting proteins of HA co-immunoprecipitation in HA-DNAJA4 SUNE1 cells by LC−MS/MS (Top20)** | | | | | | | |
| --- | --- | --- | --- | --- | --- | --- | --- |
| Accession | -10lgP | Coverage (%) IP | #Peptides | #Unique | #Spec IP | Avg. Mass | Protein name |
| **sp\|P35579\|MYH9_HUMAN** | **349.37** | **45** | **106** | **80** | **145** | **226530** | **MYH9** |
| sp\|O43795\|MYO1B_HUMAN | 291.2 | 34 | 42 | 37 | 62 | 131985 | MYO1B |
| **sp\|Q8WW22\|DNJA4_HUMAN** | **271.02** | **47** | **34** | **34** | **75** | **44798** | **DNAJA4** |
| sp\|P35580\|MYH10_HUMAN | 248.33 | 18 | 36 | 16 | 46 | 228997 | MYH10 |
| sp\|O15231\|ZN185_HUMAN | 234.98 | 40 | 24 | 24 | 33 | 73525 | ZNF185 |
| sp\|P14618\|KPYM_HUMAN | 178.52 | 26 | 11 | 11 | 12 | 57937 | PKM |
| sp\|P17844\|DDX5_HUMAN | 157.26 | 15 | 10 | 8 | 12 | 69148 | DDX5 |
| sp\|P63244\|RACK1_HUMAN | 146.7 | 27 | 8 | 8 | 8 | 35077 | RACK1 |
| sp\|P13639\|EF2_HUMAN | 140.89 | 11 | 11 | 11 | 11 | 95338 | EEF2 |
| sp\|P07355\|ANXA2_HUMAN | 140.84 | 32 | 9 | 9 | 9 | 38604 | ANXA2 |
| sp\|Q9NR30\|DDX21_HUMAN | 138.11 | 8 | 6 | 6 | 7 | 87344 | DDX21 |
| sp\|P04843\|RPN1_HUMAN | 127.96 | 12 | 7 | 7 | 8 | 68569 | RPN1 |
| sp\|Q08211\|DHX9_HUMAN | 108.61 | 5 | 5 | 5 | 5 | 140958 | DHX9 |
| sp\|P68104\|EF1A1_HUMAN | 105.2 | 11 | 6 | 6 | 6 | 50141 | EEF1A1 |
| sp\|P31689\|DNJA1_HUMAN | 99.22 | 10 | 4 | 3 | 5 | 44868 | DNAJA1 |
| **sp\|Q13200\|PSMD2_HUMAN** | **89.99** | **4** | **3** | **3** | **3** | **100200** | **PSMD2** |
| sp\|P62917\|RL8_HUMAN | 89.55 | 14 | 3 | 3 | 3 | 28025 | RPL8 |
| sp\|Q92841\|DDX17_HUMAN | 88.28 | 4 | 3 | 1 | 4 | 80273 | DDX17 |
| sp\|P25705\|ATPA_HUMAN | 87.92 | 3 | 2 | 2 | 3 | 59751 | ATP5F1A |
| sp\|P62805\|H4_HUMAN | 87.29 | 27 | 3 | 3 | 3 | 11367 | H4C1 |
